# Genome-wide association mapping for eyespot disease in US Pacific Northwest winter wheat

**DOI:** 10.1371/journal.pone.0194698

**Published:** 2018-04-02

**Authors:** Megan J. Lewien, Timothy D. Murray, Kendra L. Jernigan, Kimberly A. Garland-Campbell, Arron H. Carter

**Affiliations:** 1 Department of Crop and Soil Sciences, Washington State University, Pullman, WA, United State of America; 2 Department of Plant Pathology, Washington State University, Pullman, WA, United State of America; 3 USDA-ARS Wheat Health, Genetics, and Quality Unit, Washington State University, Pullman, WA, United State of America; Institute of Genetics and Developmental Biology Chinese Academy of Sciences, CHINA

## Abstract

Eyespot, caused by the soil-borne necrotrophic fungi *Oculimacula yallundae* and *O*. *acuformis*, is a disease of major economic significance for wheat, barley and rye. Pacific Northwest (PNW) winter wheat (*Triticum aestivum* L.) grown in areas of high rainfall and moderate winters is most vulnerable to infection. The objective of this research was to identify novel genomic regions associated with eyespot resistance in winter wheat adapted to the PNW. Two winter wheat panels of 469 and 399 lines were compiled for one of the first genome-wide association studies (GWAS) of eyespot resistance in US winter wheat germplasm. These panels were genotyped with the Infinium 9K and 90K iSelect SNP arrays. Both panels were phenotyped for disease resistance in a two-year field study and in replicated growth chamber trials. Growth chamber trials were used to evaluate the genetic resistance of *O*. *acuformis* and *O*. *yallundae* species separately. Best linear unbiased predictors (BLUPs) were calculated across all field and growth chamber environments. A total of 73 marker-trait associations (MTAs) were detected on nine different chromosomes (1A, 2A, 2B, 4A, 5A, 5B, 7A, 7B and 7D) that were significantly associated (*p-*value <0.001) with eyespot resistance in Panel A, and 19 MTAs on nine different chromosomes (1A, 1B, 2A, 2D, 3B, 5A, 5B, 7A, and 7B) in Panel B. The most significant SNPs were associated with *Pch1* and *Pch2* resistance genes on the long arms of chromosome 7D and 7A. Most of the novel MTAs appeared to have a minor effect on reducing eyespot disease. Nevertheless, eyespot disease scores decreased as the number of resistance alleles increased. Seven SNP markers, significantly associated with reducing eyespot disease across environments and in the absence and presence of *Pch1* were identified. These markers were located on chromosomes 2A (*IWB8331*), 5A (*IWB73709*), 5B (*IWB47298*), 7AS (*IWB47160*), 7B (*IWB45005*) and two SNPs (*Ex_c44379_2509* and *IAAV4340*) had unknown map positions. The additive effect of the MTAs explained most of the remaining phenotypic variation not accounted for by *Pch1* or *Pch2*. This study provides breeders with adapted germplasm and novel sources of eyespot resistance to be used in the development of superior cultivars with increased eyespot resistance.

## Introduction

Eyespot, also known as strawbreaker foot rot (*Oculimacula yallundae* and *O*. *acuformis*), is an economically important disease in wheat (*Triticum aestivum* L.) growing areas worldwide [1–2]. In the United States, it is a serious problem in the Pacific Northwest (PNW) and in areas where wheat is continuously grown and weather conditions are cool and moist. Both fungal species colonize the base of the stem, producing dark elliptical lesions and destroying structural and conductive tissue, resulting in reduced grain filling and plant lodging. When disease pressure is severe, yield losses of up to 50% have been reported in commercial fields [1], confirming the need to find effective methods for disease control. Eyespot has traditionally been controlled with fungicide application; however, fungicides may no longer be cost effective, and numerous strains are resistant to the commonly used chemicals [3–4]. Today, the use of cultivars resistant to eyespot is the most favorable control method for this destructive disease.

There are only two known sources of genetic resistance to eyespot currently used in US wheat breeding programs, *Pch1* and *Pch2*. *Pch1* was transferred from *Aegilops ventricosa* Tausch into the hexaploid wheat breeding line VPM-1 [5] as a single dominate gene mapped to the long arm of chromosome 7D [6]. While *Pch1* can significantly reduce eyespot infection, it does not confer complete resistance; furthermore, there is substantial variation in eyespot susceptibility among lines with the *Pch1* source of resistance. The second resistance gene, *Pch2*, was introduced from the French cultivar ‘Cappelle Desprez’, and acts as a single partially dominate gene [7]. *Pch2* resistance has been mapped to the distal portion of the long arm of 7A [8], and does not provide sufficient resistance under severe eyespot condition [9]. In addition, *Pch2* has been reported to be less effective against *O*. *yallundae*, the predominant strain found in the PNW, than it is to *O*. *acuformis* [2]. Consequently, additional forms of genetic resistance are necessary to improve the effectiveness and broaden the genetic diversity of eyespot resistance.

New sources of eyespot resistance have been identified in wild genetic resources including *Dasypyrum villosum* [10], *T*. *tauschii* [11], *T*. *monococcum* [12], *Thinopyrum ponticum* [13], *Th*. *intermedium* [14], and *A*. *longissima* [15]. Wild sources of resistance are not readily used in wheat breeding programs because of linkage drag and suppressed recombination [16]. Therefore, identifying novel eyespot resistance in adapted breeding material would be advantageous, as these resistance loci can be more rapidly introgressed with limited linkage drag.

Lind et al. [17] and Börner et al. [18] detected different levels of resistance in many hexaploid wheat cultivars not containing *Pch1* or *Pch2*. Additionally, Lind et al. [19] and Doussinault and Dosba [20] found genotypes that carry *Pch1*, but not *Pch2*, to have significantly different levels of resistance. These findings suggest that *Pch1* and *Pch2* are not the only sources of resistance in hexaploid wheat cultivars, or they are modified by, or interact with, other genes. New sources of eyespot resistance have been identified in European winter wheat germplasm. Burt et al. [21] discovered a QTL on chromosome 5A (*QPch*.*jic-5A*) of Cappelle Desprez that confers partial eyespot resistance. In addition, Zanke et al. [22] found several minor associations on chromosomes 1D, 1B, 2A, 2B, 2D, 3B, 3D, 5A, 5D, 6A, 7A and 7D in European winter wheat accessions.

In this study, we used two winter wheat panels consisting of released cultivars and advanced breeding material adapted for the PNW. The objectives of this research were twofold: (1) to conduct an association mapping study using genome-wide SNP markers to identify chromosome regions associated with, and gain a better genetic understanding of eyespot resistance; and (2) to determine if the resistance alleles identified in this study have an additive effect through pyramiding or if they modify or interact with the major *Pch1* resistance gene.

## Materials and methods

### Plant materials

Two winter wheat panels were used in this study to better understand the genetic control of eyespot resistance. The two panels—Panel A and Panel B—have a total of 469 and 399 winter wheat lines, respectively, including breeding lines (90% of the panels) and released cultivars (10% of the panels) mainly from PNW breeding programs. Breeding lines varied in filial generation, with F_3:5_ lines being the earliest generation in the panel. Panel A includes only soft white winter wheat accessions with either a club or lax head type. Panel B includes accessions from five market classes including soft white (72.1%), club (20.6%), hard red (15.7%), hard white (11.6%) and soft red (0.6%) winter wheat. Additionally, for all field and greenhouse evaluations, susceptible, moderately susceptible and resistant wheat cultivars were used as controls. Winter wheat cultivars ‘Madsen’ (PI 511673) [23] and ‘Cara’ (PI 643435) [24], both containing *Pch1*, were used as resistant controls. Cappelle Desprez, a French cultivar containing *Pch2*, was used as a moderately resistant control. The susceptible controls used were ‘Hill 81’ (Cltr 17954) and ‘Eltan’ (PI 536994) [25].

### Field evaluation

Panels A and B were evaluated for eyespot resistance in a total of three year-location environments from 2014 to 2015. These locations included Washington State University (WSU) Spillman Agronomy Farm (SP) and WSU Cook Agronomy Farm (C), both located near Pullman, WA. Washington State University owns these research farms and provided the authority to plant research trials at these farms. These locations did not involve any endangered or protected species. All trails were planted in the fall and were maintained to regional commercial production practices, excluding any fungicide or herbicide treatments. All accessions were planted in 1 m rows, using 5 g of seed, and spaced 35 cm apart. Both panels were planted in an augmented randomized complete block design, unreplicated in 2014 (SP2014) and replicated, by location, in 2015 (SP2015 and C2015). Susceptible and resistant controls (susceptible Hill 81 and Eltan; resistant Cara and Madsen) were planted every 50 rows to monitor disease pressure. Field sites were selected based on previous history and crop rotation to increase eyespot infection. In addition to natural inoculum, oat (*Avena sativa* L.) kernels colonized by the eyespot pathogens were placed in each planting tray to ensure good disease pressure, for all trials. The oat kernel inoculum was created by autoclaving oat kernels and inoculating them separately with one of three PNW isolates of *O*. *yallundae*. The isolates were grown in containers for one to two months and then combined when they were added to the field trays. In 2015, at the Cook field site, both eyespot spores and infested kernels were used; spore inoculum was produced and inoculated as described by Wetzel et al. [26].

During early July, after anthesis, ten plants were randomly selected, dug up from the middle of each row, and scored per the methods of Wetzel and Murray [27]. Eyespot disease severity was determined by rating stem bases 1–2 internodes above the crown using a 0 to 4 scale, where 0 = no visual symptoms (healthy plant), 1 = less than 25% of stem circumference covered with lesions, 2 = less than 50% stem circumference colonized, 3 = less than 75% stem circumference colonized and 4 = lesion covering almost 100% girdling the base (almost dead).

### Growth chamber evaluation

Both panels were screened for eyespot resistance by inoculating them with *O*. *yallundae* and *O*. *acuformis* pathogens in separate growth chamber experiments. All growth chamber experiments were replicated twice and arranged in a randomized complete block (RCB) design. Seeds were planted into 72 cell seedling trays (cells 1-1/2" ×1-1/2" × 2.25" deep) with commercial Sunshine Potting mix (SunGro Horticulture, Bellevue, WA) and fertilized with Osmocote (14-14-14, w/v) (The Scotts Company LLC, Marysville, OH). Two seeds were planted in each cell to ensure emergence and even pathogen inoculation. Susceptible (Hill 81 and Eltan), moderately resistant (Cappelle Desprez-*Pch2*), and resistant (Cara-*Pch1* and Madsen-*Pch1*) controls were planted in each tray to monitor disease pressure. The trays were first placed in growth chambers at 15°C day/12°C night, with a 12 h photoperiod and 100% relative humidity. Once plants reached the two-leaf stage, temperature was reduced to 12°C day/night, and the mist function was turned on to ensure high humidity (5 s mist every 5 min). The mist function remained on until visual ratings were conducted.

Plants were inoculated at the two-leaf stage following the procedure described by Sheng and Murray [15]. In all experiments either four *O*. *yallundae* or four *O*. *acuformis* isolates were used; however, unlike Sheng and Murray [15], the isolates were not GUS transformed. Eight to ten weeks after inoculation visual disease ratings were conducted. Rating was done on a 0 to 4 scale [15] where 0 = no symptoms (healthy), 1 = a lesion only on the first leaf sheath, 2 = a lesion on the first leaf sheath and a small lesion on the second leaf sheath, 3 = a lesion covering the first leaf sheath and up to half of the second sheath and 4 = a lesion covering the first and second sheaths (nearly dead). Each line was given a score based on the visual rating of two plants.

### DNA extraction and genotyping

Genomic DNA was extracted from juvenile leaf tissue using the BioSprint 96 DNA Plant Kit (QIAGEN, Hilden, Germany). Genotyping was carried out at the USDA-ARS genotyping laboratory at Fargo, ND using the Infinium 90K (Panel A) and 9K (Panel B) iSelect SNP arrays from the Illumina platform (Illumina Inc., San Diego, CA) described by Wang et al. [28] and Cavanagh et al. [29]. The raw Illumina SNP data were visualized, manipulated and filtered in GenomeStudio v2011.1 software (Illumina, Inc.). Chromosome and chromosome location for the 90K SNP and 9K SNP markers were based on the maps developed by Wang et al. [28] and Cavanagh et al. [29]. Markers that were monomorphic or had missing values > 20% were removed from the data set. The remaining missing marker data was imputed using BEAGLE v4.1 with default parameters [30]. After processing, 28,779 SNPs remained for Panel A (24,557 mapped SNPs), and 6,783 SNPs (6,359 mapped markers) remained for Panel B. As Panel A was genotyped on the 90K SNP array it had over four times more markers than Panel B, which was run on the 9K array. For both panels, the SNPs were distributed evenly across all chromosomes, though the D genome had 50 to 80% less coverage than the A or B genomes (S1 Table). Marker analysis for *Pch1* was conducted using the simple sequence repeat markers *Xorw1* and *Xorw5* [31] and the KASP assay wMAS0000023 [32] following the protocol of LGC Genomics (http://www.lgcgenomics.com/).

### Statistical analysis

Data analysis was performed using the R statistical software [33]. For all field experiments each year-location was considered as an environment totaling three field environments (SP2014, SP2015, and C2015) with the ten subsamples averaged to provide a mean entry score for each environment. In the growth chamber experiments the *O*. *yallundae* and *O*. *acuformis* species were analyzed separately and replications for each species were averaged, totaling two growth chamber environments (GC_OY and GC_OA). A best linear unbiased predictor (BLUP) value for each entry was calculated using entry mean scores from all five environments using the lme4 package [34]. BLUP values were also calculated for all individual field and growth chamber environments to aid in normalizing the data and were used in the association analysis. Pearson product momentum correlations using entry means for the five locations were calculated using the cor.test function in R. The broad sense heritability (H^2^) for all field and growth chamber environments was estimated for all traits according to the following formula:

H2=σ2G/[(σ2G+(σ2GE/e)+(σ2/e)]

where σ^2^G is variance of genotype, σ^2^GE is variance of genotype-by-environment interaction; e is number of environment; and σ^2^ is within environment error variance.

### Genome-wide association analysis

Genome-wide association analysis was conducted using the compressed mixed linear model (CMLM) implemented in the GAPIT R package [35–36]. The CMLM approach accounts for population structure (Q) and relatedness among individuals [kinship matrix (K)] to reduce false discoveries [37]. The K and Q was calculated by principle component analysis (PCA) and the VanRaden method [38] implemented in GAPIT using marker data imported for mapping. Marker-trait associations (MTAs) were only declared significant if they had a minor allele frequency (MAF) greater than 0.05, nominal *p*-values (*p* ≤ 0.001), and were found significant in at least two environments or significant for the combined environment BLUP score.

Linkage disequilibrium (LD) (r^2^) was calculated between all significant MTAs found on the same chromosome in order to identify linked markers using GGT 2.0 [39]. Markers were declared linked if LD had an *r*^*2*^ value greater than or equal to 0.2. If the LD grouping was unclear, markers were declared in linkage if greater than or equal to 15 cM apart. Within the linked MTAs the marker with the strongest association with eyespot resistance was selected as the representative marker (tagging marker) based on the phenotypic variation explained by marker, the allelic effect, the FDR adjusted and nominal *p*-values, and the number of environments in which a significant MTA was found.

As the 90K and 9K SNP arrays were used to genotype Panels A and B, respectively, only the 90K consensus map positions were reported as most of the significant markers identified in Panel B (9K array) were present in the 90K consensus map. If a significant marker from Panel B (9K array) was not present in the 90K consensus map the next most significant and linked marker with a 90K map position was used. Using only the 90K consensus map allowed for the identification of common genomic regions associated with eyespot between panels.

To assess the breeding utility of the MTAs identified in this study, the effect of pyramiding the SNP haplotypes associated with eyespot resistance was tested. Haplotype data from the major tagging markers were used along with the *Pch1* marker data to determine the number of eyespot resistance alleles carried by each line. The number of cumulative resistance alleles was then regressed against the BLUP score for all five environments combined using the lm function in R. All lines not containing the *Pch1* resistance allele were used in a second linear regression analysis to more clearly see the effect of the minor MTAs.

## Results

### Field phenotypic data

**Fig 1:**
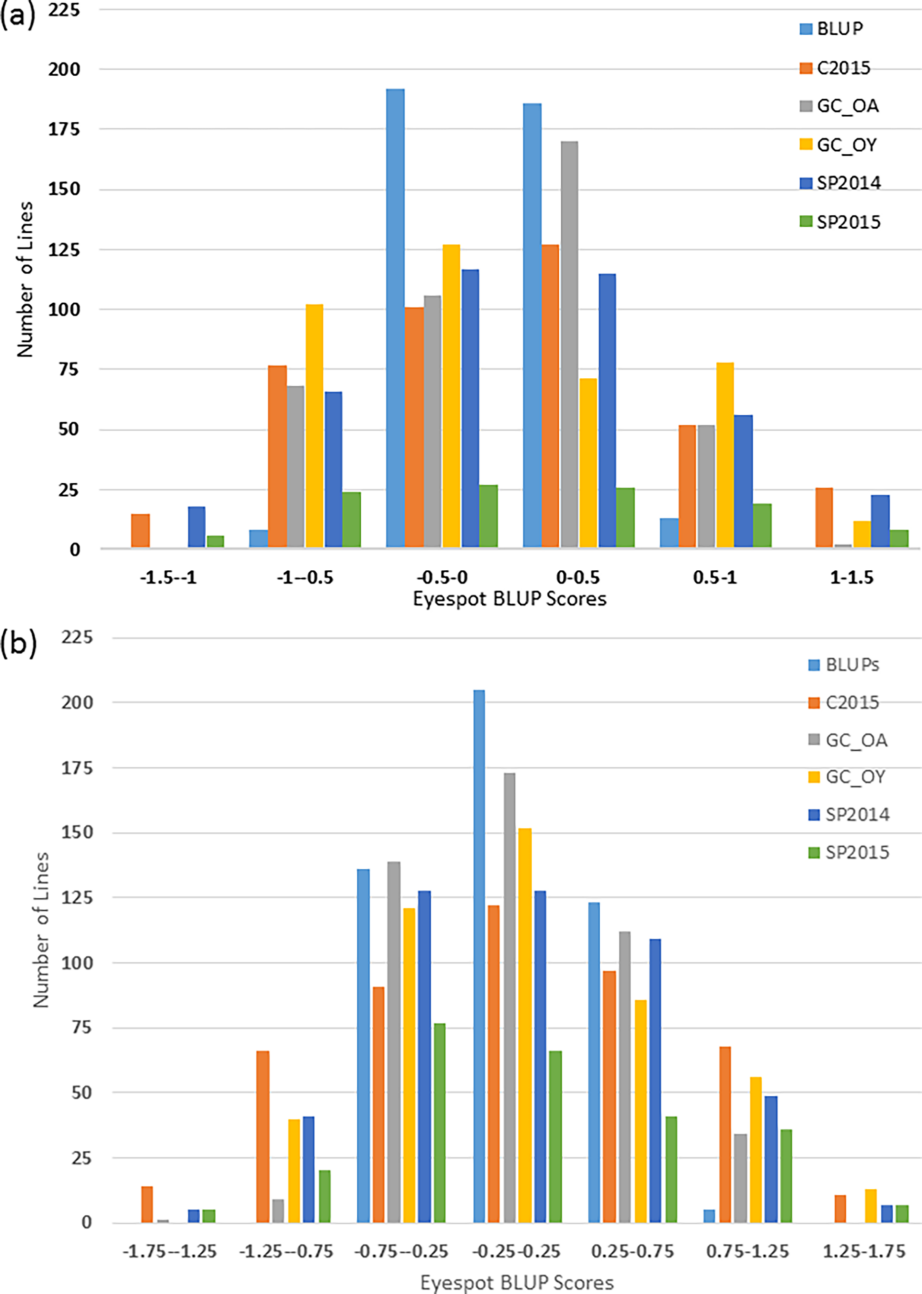
Frequency distribution of eyespot scores for all five environments and BLUPs in winter wheat (a) Panel A (469 lines) and (b) Panel B (399 lines). Field locations included Washington State University (WSU) Spillman Agronomy Farm (SP) in 2014 and 2015, and WSU Cook Agronomy Farm (C) in 2015 only, both located near Pullman, WA. Growth chamber environments were separated by species *O*. *acuformis* and *O*. *yullundae* (GC_OA and GC_OY).

**Fig 2:**
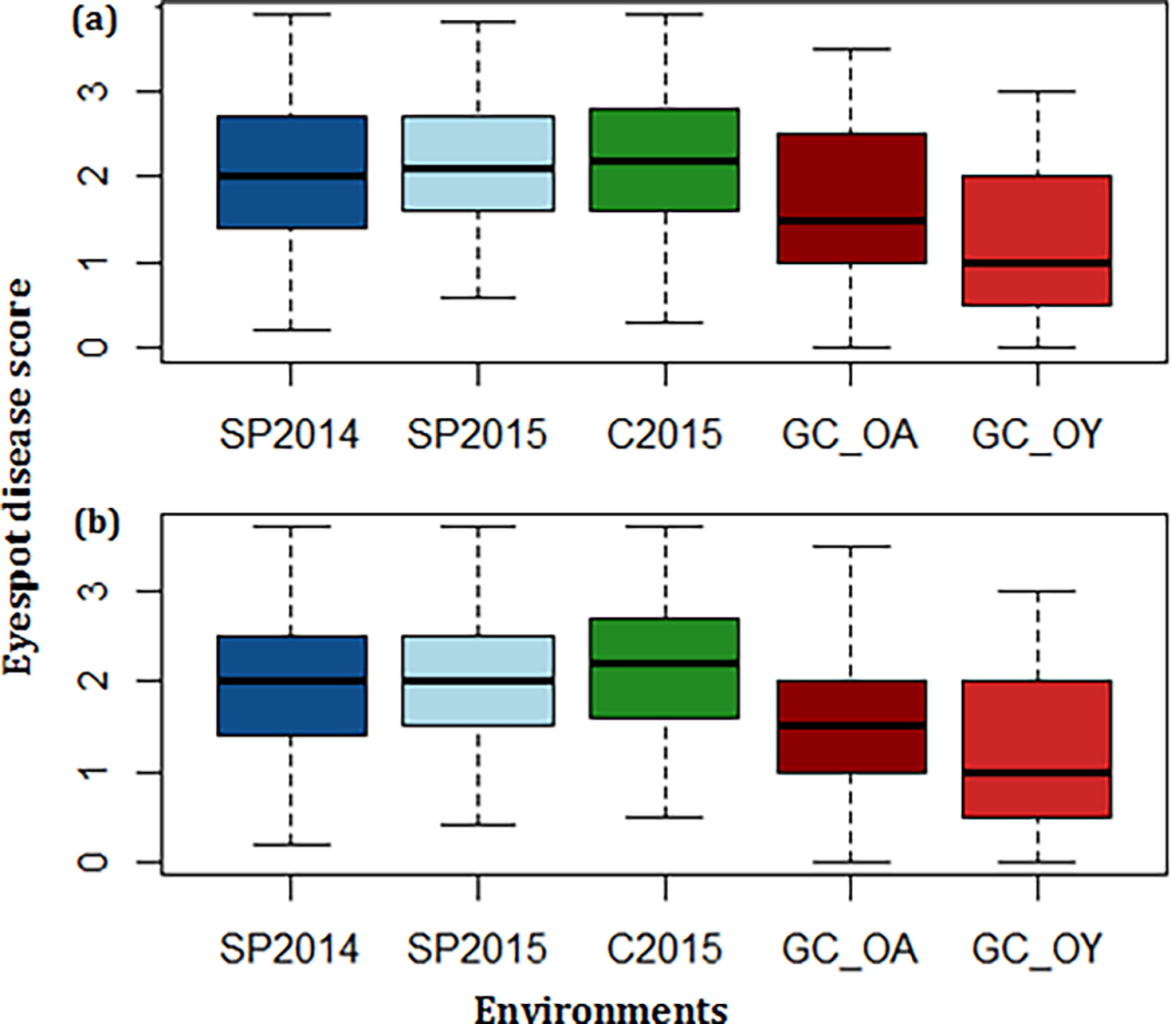
Distribution of mean eyespot disease scores for all five individual environments in winter wheat Panel A (a) and Panel B (b). Panels A and B were evaluated for eyespot resistance in a total of three field environments from 2014 to 2015, and two growth chamber (GC) environments. Field locations included Washington State University (WSU) Spillman Agronomy Farm (SP) and WSU Cook Agronomy Farm (C), both located near Pullman, WA. Growth chamber environments were separated by species *O*. *acuformis* and *O*. *yullundae* (GC_OA and GC_OY).

Disease pressure for the Spillman farm 2014 (SP2014) and the Cook farm 2015 (C2015) field environments was strong, where the susceptible checks Eltan and Hill 81 were rated as highly susceptible (3–4). The Spillman 2015 (SP2015) field environment had strong disease pressure; however, it is important to note that there was extensive weed pressure resulting in significant amount of missing data. For all field environments, the phenotypic data appeared to be near normal (Fig 1). The mean disease scores for all field environments ranged from 0.20 to 3.90 for Panel A and 0.20 to 3.70 for Panel B, with mean disease scores of 2.00 for both panels (Fig 2). The disease scores of all individual field environments have correlation coefficients ranging from 0.481 to 0.833 for Panel A and 0.169 to 0.806 for Panel B (S2 Table). Field heritability was 0.610 and 0.467 for Panels A and B, respectively. The moderate correlation between environments and medium field heritability was expected as eyespot is a difficult disease to phenotype in the field and environmental factors highly impact pathogen virulence.

### Growth chamber phenotypic data

In all growth chamber evaluations, the susceptible checks Hill 81 and Eltan scored 3–4, the moderately resistant check Cappelle Desprez scored 0–2, and the resistant checks Madsen and Cara scored 0–1. Phenotypic data was not normal for Panels A or B; therefore, BLUP values were calculated for both growth chamber environments, resulting in a more normal distribution (Fig 1). The growth chamber disease scores for *O*. *acuformis* (GC_OA) in Panels A and B ranged from 0.00 to 3.50 with mean disease scores of 1.50 for both panels (Fig 2). *O*. *yallundae* (GC_OY) disease scores for Panels A and B ranged from 0.00 to 3.00 with mean disease scores of 1.00. The heritability in the growth chamber was 0.79 and 0.82 for Panels A and B, respectively. The GC_OA and GC_OY environments had a high correlation coefficient of 0.741 for Panel A and 0.707 for Panel B (S2 Table).

### BLUPs

A BLUP value for each line was calculated using mean scores from all five environments. These values ranged from -0.71 to 0.97 for Panel A, and -0.60 to 0.67 for Panel B with mean scores of 0.01 (negative values indicated a reduction in eyespot disease). The values appeared to have a normal distribution (Fig 1) and were highly correlated with all environments (S2 Table).

### Genotyping and *Pch1* marker results

In Panel A, after filtering for 20% missing data and removing markers which were monomorphic, a total of 28,779 SNP markers were used for analysis. Of these markers, 2,497 had a least one line heterozygous, or about 9% of the markers showing heterozygosity. The levels of heterozygosity varied within these markers, ranging from 46 to 0.2%. Overall, the level of heterozygosity in Panel A was estimated to be 1%. In Panel B, after filtering for missing data and removing monomorphic markers, 6,783 markers remained for analysis. Only 713 markers in this Panel B showed heterozygosity, representing about 11% of the markers. The overall level of heterozygosity in Panel B was 5%, and ranged from 24 to 2% per individual marker.

*Pch1* marker results showed that 54% of Panel A’s lines had the *Pch1* resistance allele, 35% were null for *Pch1* and 11% heterozygous. Forty-two percent of Panel B’s lines had the *Pch1* resistance allele, 36% were null for *Pch1* and 22% heterozygous. The level of heterozygosity for this marker in particular is higher than the average amount of heterozygosity in the panels as a whole. Breeding programs in the PNW intentionally cross *Pch1* donors with susceptible lines to incorporate resistance into the breeding program [40–42]. These donor lines are oftentimes genetically related to the susceptible lines they are crossed to [42]. Thus, breeding lines segregate at the *Pch1* locus, but are oftentimes fixed at other important loci throughout the genome. This breeding strategy may demonstrate an elevated level of heterozygosity at the *Pch1* locus but not at other loci. Additionally, lower yield has been associated with incorporation of the *Pch1* gene into susceptible germplasm [43]. Many breeders looking phenotypically at segregating populations may inadvertently select for heterozygosity at the *Pch1* locus, which can show moderate levels of resistance with limited yield penalties.

**Fig 3:**
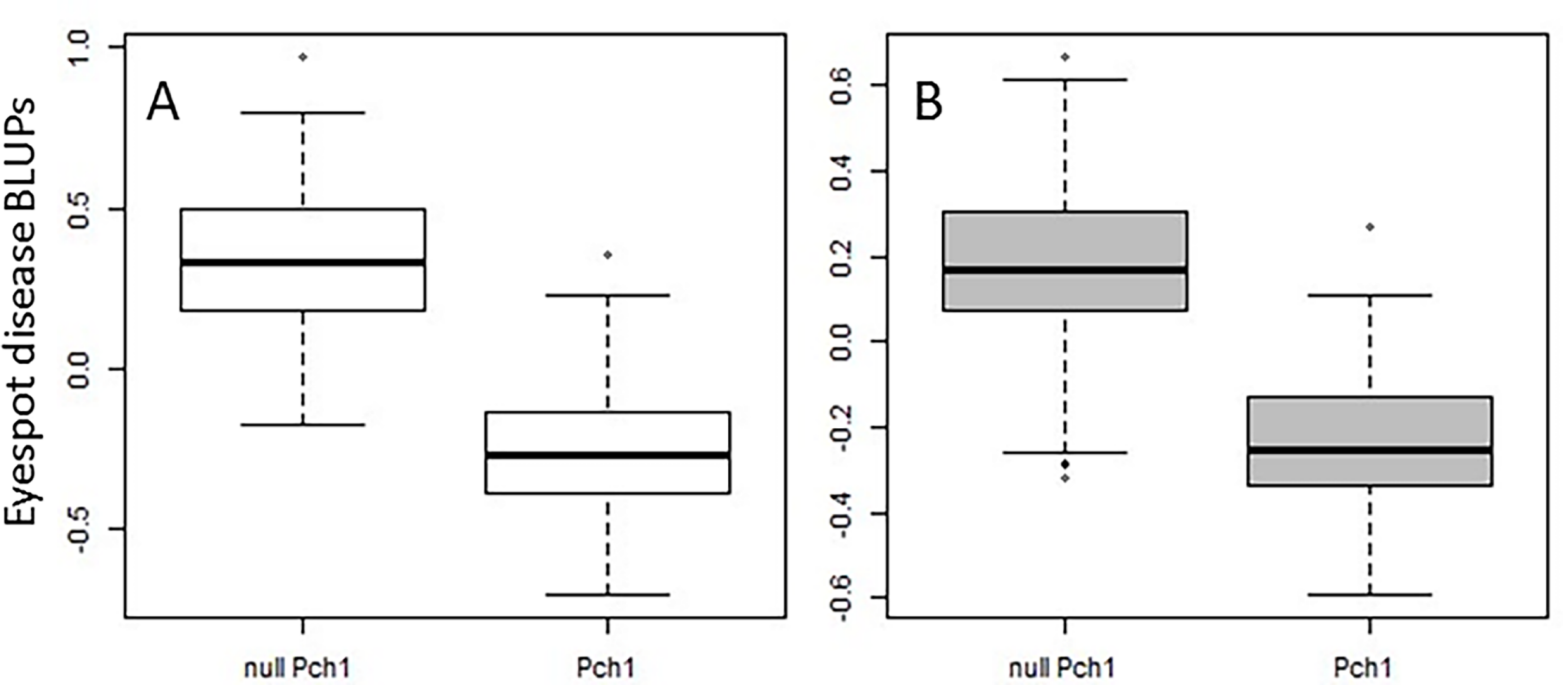
Boxplots of eyespot disease BLUPs for all winter wheat lines without *Pch1* (null *Pch1)* and all lines with *Pch1* resistance allele (*Pch1)* in Panel A (A) and Panel B (B).

For all lines with the *Pch1* resistance allele BLUP values ranged from -0.71 to 0.59 for Panel A and -0.60 to 0.27 for Panel B with mean disease scores of approximately -0.25 (Fig 3). BLUPs for lines without *Pch1* ranged from -0.18 to 0.97 for Panel A and -0.29 to 0.67 for Panel B with mean scores of 0.33 and 0.19, respectively (Fig 3). While the disease score for lines without *Pch1* were significantly higher than lines with *Pch1* for all environments, there were a substantial number or lines without *Pch1* that exhibited moderate to high resistance (BLUPs ≤ 0.2).

### Principle component analysis

Principle component analysis (PCA) was conducted to identify and adjust for the population structure found in both panels. In Panel B three main subgroups were identified using PC1 and PC2 [44]. The genotypes were grouped by market class, with significant overlap between groups. The first group was made up of club wheat (*T*. *aestivum* spp. *compactum*) lines. The second group was composed of common wheat (*T*. *aestivum* spp. *aestivum*), which was further subdivided into hard red and soft white market classes [44]. Principle component analysis of Panel A revealed two main subgroups identified by PC1 and PC2 (S1 Fig) [45]. These subgroups, like Panel B, were also grouped by market class with some overlap between groups. However, unlike Panel B, Panel A did not contain lines in the hard red market class.

### Association analysis

Genome-wide association analysis of eyespot disease for all individual field and growth chamber environments and BLUPs detected 73 SNP markers on nine different chromosomes (1A, 2A, 2B, 4A, 5A, 5B, 7A, 7B and 7D) in Panel A (S3 Table) and 19 significant markers on nine different chromosomes (1A, 1B, 2A, 2D, 3B, 5A, 5B, 7A and 7B) in Panel B (S3 Table) that were significantly associated (*p*-value <0.001) with eyespot resistance. In addition, twelve significant markers with unknown map positions were detected for Panel A and three significant markers with unknown map positions were detected for Panel B.

The most significant MTAs detected in Panel A were on the long arms of chromosomes 7A and 7D, where *Pch2* and *Pch1* are positioned, respectively. The marker of greatest significance on 7A, *IWB41099*_*(A)*_, and presumed to be linked to *Pch2*, was positioned at 241.1 cM and had allelic effect estimates ranging from -0.14 to -0.26 and *R*^*2*^ values from 0.03 to 0.06. In Panel B only one MTA was detected on 7A (*IWA8312*_*(B)*_) located at 171.1 cM. In both populations, the markers liked to *Pch2* on chromosome 7A had minor allele frequencies of ≤ 0.07 (32 entries). In contrast, the *Pch1* linked SSR and KASP markers (*Xorw1*, *Xorw5*, and *Pch1*) had strong allele effect estimates (-0.30 to -0.55) and *R*^*2*^ values from 0.12 to 0.18. Therefore, owing to the low frequency of the SNP markers on chromosome 7A presumed to be linked to *Pch2* and their low *R*^*2*^ values, and the lack of a DNA test for *Pch2*, only *Pch1* resistance was accounted for when evaluating the effect of other loci on eyespot resistance.

As expected, the most significant MTAs detected in Panel A and Panel B were on the long arms of chromosomes 7A and 7D (Panel A only), where *Pch2* and *Pch1* are presumed to be positioned. Although *Pch1* has been used extensively in the Pacific Northwest breeding programs, *Pch2* has been used less frequently. There may be two reasons why *Pch2* has been used less frequently in US germplasm than other parts of the world. First, *Pch2* has been reported to be less effective against *O*. *yallundae* than *O*. *acuformis* [2]. Second, there are few diagnostic markers associated with tracking *Pch2* in winter wheat germplasm, making it more difficult to confirm presence of *Pch2*, especially in the presence of *Pch1*. The MTA identified on chromosome 7A is likely linked to *Pch2*. The MTA on chromosome 7A is in the same genetic region as *Pch2* is located. Only 32 entries in the panels tested carried the resistance allele on chromosome 7A. Pedigree analysis of the 32 entries carrying the 7A locus indicated many of these lines contained European ancestry, of which many are reported to contain *Pch2*. Interestingly, the MTAs on chromosome 7A were detected in four of the six environments, including those of both *O*. *yallundae* and *O*. *acuformis*. This is of importance because previous studies reported *Pch2* to be less effective against O. *yallundae* than *O*. *acuformis* [2]. Therefore, in the germplasm used in this study, the effect of the MTA on chromosome 7A (presumed to be linked to *Pch2*) cannot be assumed to be identical to that reported previously in different germplasm sets. Further investigation is ongoing to confirm if this region is indeed *Pch2*, but may give breeders a place to start when pyramiding effective resistance into lines already containing *Pch1*.

Excluding markers with unknown map position, markers linked to *Pch1* and potentially linked with *Pch2*, twenty unique loci were identified between the two panels. The phenotypic variation explained (*R*^*2*^) by the significant markers ranged from 0.01 to 0.15 for Panel A and 0.02 to 0.05 for Panel B (Tables 1 and 2).

**Table 1:** Genome-wide association mapping (GWAS) analysis results for eyespot disease resistance in winter wheat Panel A. Markers associated with eyespot disease resistance with a *p*-value ≤ 0.005 and identified in two or more environments including BLUPS, are reported.

SNP^a^	Chr^b^	cM^c^	Minor		Environments^e^					
* *			allele^d^		BLUPs	SP2014	SP2015	C2015	GC_OA	GC_OY
***IWB8331***	2A	101.97	G	*p*^*f*^	1.13E-03	-	-	1.72E-03	-	-
*IWB43628*				*R*^*2*^^*g*^	0.01	-	-	0.02	-	-
*IWA3569*				*AE*^*h*^	-0.121	-	-	-0.157	-	-
***IWB73709***	5A	89.02	T	*p*	1.94E-03	-	-	-	-	-
				*R*^*2*^	0.01	-	-	-	-	-
* *				*AE*	0.107	-	-	-	-	-
***IWB47298***	5B	100.64	T	*p*	4.95E-04	2.04E-03	-	-	1.20E-03	-
				*R*^*2*^	0.01	0.02	-	-	0.02	-
* *				*AE*	-0.080	-0.127	-	-	-0.112	-
***IWB47160***	7AS	126.40	T	*p*	-	-	-	-	5.95E-04	1.18E-03
*IWB49474*				*R*^*2*^	-	-	-	-	0.02	0.01
* *				*AE*	-	-	-	-	-0.151	-0.186
***IWB45005***	7BL	158.98	T	*p*	5.41E-18	9.18E-10	-	2.82E-12	9.40E-19	2.18E-12
*IWB9330*				*R*^*2*^	0.09	0.07	-	0.09	0.12	0.07
* *				*AE*	-0.187	-0.225	-	-0.320	-0.285	-0.289
***IWB20731***	-	-	G	*p*	8.53E-04	-	-	-	-	7.59E-04
				*R*^*2*^	0.01	-	-	-	-	0.02
				*AE*	-0.084	-	-	-	-	-0.164
***IWB32948***	-	-	G	*p*	3.70E-04	-	-	-	3.23E-04	4.68E-04
				*R*^*2*^	0.01	-	-	-	0.02	0.02
* *				*AE*	0.103	-	-	-	0.152	0.193

^a^ Underlined and bolded markers indicate the ‘most significant tagging marker’

^b^ Chromosomal location; ‘-’ indicates unmapped SNPs that were significant in this analysis

^c^ Chromosome postion according to Wang et al. (2014)

^d^ Allele that the allelic effect estimate (AE) is in respect to

^e^ Spillman 2014 and 2015 (SP2014, SP2015), Cook 2015 (C2015), Best Linear Unbiased Predictions (BLUPs), and growth chamber *O*. *acuformis* and *O*. *yullundae* (GC_OA, GC_OY)

^f^ *p* indicates the significance of SNP marker

^g^ R^2^ indicates phenotypic variation explained by significant SNP

^h^ AE is the allelic effect estimate in respect to the minor allele

**Table 2:** Genome-wide association mapping analysis (GWAS) results for eyespot disease resistance in winter wheat Panel B. Markers associated with eyespot disease resistance with a *p*-value ≤ 0.005 and identified in two or more environments including BLUPS, are reported.

SNP^a^	cM^b^	Chr.^c^	Minor		Environments^e^					
* *			allele^d^		BLUPs	SP2014	SP2015	C2015	GC_OA	GC_OY
***IWA5505***	1AL	118.27	G	*p*^*f*^	3.96E-03	6.63E-04	-	-	-	-
*IWA4934*				*R*^*2*^^*g*^	0.02	0.03	-	-	-	-
				*AE*^*h*^	-0.063	-0.261	-	-	-	-
***IWA4897***	1AL	137.20	T	*p*	5.41E-04	1.38E-04	-	-	-	-
				*R*^*2*^	0.03	0.03	-	-	-	-
* *				*AE*	-0.137	-0.149	-	-	-	-
***IWA4089***	1BL	78.45	G	*p*	9.92E-04	-	8.93E-06	-	2.16E-04	1.37E-04
				*R*^*2*^	0.02	-	0.04	-	0.03	0.03
* *				*AE*	-0.111	-	-0.212	-	-0.212	-0.286
***IWA5161***	2A	167.87	G	*p*	8.97E-06	8.61E-05	-	1.87E-03	5.10E-04	9.75E-04
				*R*^*2*^	0.04	0.03	-	0.02	0.02	0.02
* *				*AE*	-0.068	-0.042	-	-0.108	-0.089	-0.070
***IWA551***	2DL	98.59	T	*p*	3.13E-04	-	-	-	1.34E-03	1.42E-03
*IWA5894*				*R*^*2*^	0.03	-	-	-	0.02	0.02
* *				*AE*	0.096	-	-	-	0.119	0.186
***IWA4054***	3B	62.57	C	*p*	-	-	-	-	1.67E-03	1.40E-03
				*R*^*2*^	-	-	-	-	0.02	0.02
				*AE*	-	-	-	-	-0.136	-0.114
***IWA8203***	3B	144.74	T	p	-	-	-	-	5.12E-06	7.01E-04
*IWA2147*				*R*^*2*^	-	-	-	-	0.04	0.03
* *				AE	-	-	-	-	-0.126	-0.121
***IWA5923***	5A	15.86	G	*p*	1.46E-03	1.83E-03	-	-	-	-
*IWA3567*				*R*^*2*^	0.02	0.02	-	-	-	-
				*AE*	0.068	0.046	-	-	-	-
***IWA1***	5AL	90.54	G	p	-	-	-	1.39E-03	-	1.26E-03
*IWA4719*				*R*^*2*^	-	-	-	0.02	-	0.02
* *				AE	-	-	-	-0.276	-	-0.288
***IWA7708***	5B	150.93	G	*p*	1.07E-03	-	-	-	-	-
				*R*^*2*^	0.02	-	-	-	-	-
* *				*AE*	-0.052	-	-	-	-	-
***IWA598***	7B	142.24	G	*p*	1.26E-06	5.24E-04	-	5.04E-04	2.36E-05	1.65E-05
				*R*^*2*^	0.05	0.03	-	0.03	0.04	0.04
* *				*AE*	-0.087	-0.148	-	-0.043	-0.106	-0.170
***IWA505***	-	-	G	*p*	-	-	-	-	3.01E-04	4.84E-04
				*R*^*2*^	-	-	-	-	0.03	0.02
				*AE*	-	-	-	-	0.113	0.089
***IWA4046***	-	-	C	*p*		-	-	-	4.78E-04	1.59E-03
				*R*^*2*^	-	-	-	-	0.03	0.02
				*AE*	-	-	-	-	-0.292	-0.172
***IWA2226***	-	-	G	*p*	1.65E-03	-	-	-	1.48E-03	4.91E-04
				*R*^*2*^	0.02	-	-	-	0.02	0.03
* *				*AE*	-0.067	-	-	-	-0.094	-0.162

^a^ Underlined and bolded markers indicate the ‘most significant tagging marker’

^b^ Chromosomal location; ‘-’ indicates unmapped SNPs that were significant in this analysis

^c^ Chromosome postion according to Wang et al. (2014)

^d^ Allele that the allelic effect estimate (AE) is in respect to

^e^ Spillman 2014 and 2015 (SP2014, SP2015), Cook 2015 (C2015), Best Linear Unbiased Predictions (BLUPs), and growth chamber *O*. *acuformis* and *O*. *yullundae* (GC_OA, GC_OY)

^f^ *p* indicates the significance of SNP marker

^g^ R^2^ indicates phenotypic variation explained by significant SNP

^h^ AE is the allelic effect estimate in respect to the minor allele

Several MTAs were only found in the growth chamber environments. In Panel A, these markers included *IWB7149*, *IWB47345* and *IWB47160* on chromosomes 2AL, 4AS and 7AS, respectively. In Panel B these markers included *IWA4054* and *IWA8203* on chromosome 3B and unmapped markers *IWA505* and *IWA4046*. Interestingly, if markers were only detected with significance to only the greenhouse trials, the markers were significantly associated to both *O*. *yallundae* and *O*. *acuformis*. No significant markers were found to be only associated with field environments. Significant SNP markers detected in Panel A were cross-checked with the GWAS results of Panel B. While no markers were found to be significant in both panels, we did find several shared genomic regions of significance, including the long arms of chromosomes 1A, 5A, 7A and 7B.

### Evaluating the effect of MTAs

To more closely assess the effect of the MTAs discovered in this study additional analyses were run on all lines not carrying the *Pch1* resistance allele and on all lines carrying *Pch1*. Seven SNP markers, significantly associated with reducing eyespot disease across environments and in the absence and presence of *Pch1* (S2 Fig), were identified. These markers were located on chromosomes 2A (*IWB8331*), 5A (*IWB73709*), 5B (*IWB47298*), 7AS (*IWB47160*), 7B (*IWB45005*) and two SNPs (*Ex_c44379_2509* and *IAAV4340*) had unknown map positions, and are detailed below (Tables 1 and 2).

On chromosome 2A, five significant MTAs (*IWB8331*_*(A)*_, *IWB4328*_*(A)*_, *IWA359*_*(A)*_, *IWB2840*_*(A)*_, and *IWB1079*_*(A)*_) were positioned from 102.3 to 106.3 cM on 2A and were detected in two environments. In addition, two significant markers (*IWB71497*_*(A)*_ and *IWA5161*_*(B)*_) were located on the long arm of 2A positioned at 141.7 cM and 167.9 cM, respectively. The most significant tagging marker on 2A (*IWB8331*) was detected in two environments and had allelic effect estimates ranging from -0.12 to -0.15 and *R*^*2*^ values from 0.012 to 0.016. When assessing effectiveness of this marker, in both the absence and presence of *Pch1*, we found that it caused a significant (*p* ≤ 0.005) reduction in disease response (S2 Fig). This region is of specific interest as Zanke et al. [22] also detected a significant MTA for eyespot resistance (*Ra_c21740_341*) in this region. Given this region has been reported in different populations to confer resistance to eyespot, it could be an area of focus for more research to better understand the genetics of eyespot resistance in wheat.

Seven SNPs were detected on chromosome 5A with two SNPs (*IWA5923*_*(B)*_ and *IWA3567*_*(B)*_) positioned on the short arm at 15.9 cM, three SNPs (*IWB73709*_*(A)*_, *IWA1*_*(B)*_ and *IWA4719*_*(B)*_) located from 89.0 to 90.5 cM, and two more SNPs (*IWA3391*_*(A)*_ and *IWB59054*_*(A)*_) at 129.9 cM. The tagging marker *IWB73709* was found to have a significant (*p* ≤ 0.001) effect when analyzing the population as a whole; however, it was not found to significantly reduce eyespot infection in either the absence or presence of *Pch1* (S2 Fig). Of the five SNPs detected on the short arm of chromosome 5A, SNPs *IWB73709*, *IWA1*, and *IWA4719* appear to be near two SSR markers, *Xgwm639* and *Xgwm156*, reported by both Burt et al. [21] and Zanke et al. [22] to be associated with eyespot resistance. In addition, Zanke et al. [22] also reported a significant MTA with Xb*arc0303*, on the short arm of 5A near the SNPs *IWA5923* and *IWA3567* detected in this population. The germplasm used by Zanke et al. [22] consisted only of European winter wheat cultivars, and Burt et al. [21] identified 5A resistance in the European cultivar Cappelle Desprez. Even though the SNP markers presented here did not significantly reduce eyespot resistance, this region warrants further investigation given the overlap between the three studies.

On chromosome 5B eight novel MTAs were identified, with seven SNPs (*IWB34332*_*(A)*_, *IWB47298*_*(A)*_, *IWA6671*_*(A)*_, *IWB14635*_*(A)*_, *IWB40925*_*(A)*_, *IWB40926*_*(A)*_, and *IWB25936*_*(A)*_) in the positional range 100.6 to 104.6 cM, and one SNP (*IWA7708*_*(B)*_) positioned at 150.9cM. The tagging marker *IWB47298* was detected in three environments, with allelic effect estimates ranging from -0.08 to -0.13 and *R*^*2*^ values from 0.014 to 0.016. This marker was found to have a moderately significant (*p* ≤ 0.01) effect on reducing eyespot infection in the absence and presence of *Pch1* (S2 Fig).

Four significant MTAs (*IWB10471*_*(A)*_, *IWB47160*_*(A)*_, *IWB49474*_*(A)*_, and*IWB34932*_*(A)*_) on the short arm of chromosome 7A located from 126.4 to 130.3 cM were detected. This region was identified in both the growth chamber (GC_OY and GC_OA) and BLUP environments. Tagging marker *IWB47160* had allelic effect values ranging from -0.16 to -0.19, but had relatively low *R*^*2*^ values of 0.010 to 0.012. This marker was found to have a significant *(p* < 0.001) effect on reducing eyespot infection in the absence of *Pch1* and moderately significant (*p* < 0.01) effect in the presence of *Pch1* (S2 Fig). Zanke et al. [22] reported a significant MTA, *Xwmc0488b-137*, on the short arm of chromosome 7A, which appears to be near the MTA we detected using SNP marker *IWB47160*. Even though *Pch2* is known to reside on the long arm of chromosome 7A, this MTA is located on the short arm of the chromosome. The MTA significantly reduced eyespot infection in the absence of *Pch1*, as well as in combination. Given the ability to do this, and the overlap in genetic region published previously, this MTA should prove useful in breeding programs to enhance eyespot resistance.

Thirteen MTAs were located on chromosome 7B at positions ranging from 140.0 to 167.3cM, and all were found significant with either five or all six environments. The most significant marker (*IWB45005*) was located at 158.9 cM and had sizable allelic effects ranging from -0.19 to -0.32 and relatively high *R*^*2*^ values from 0.07 to 0.12, the highest observed outside of *Pch1*. This marker was found to have a significant (*p* < 0.001) effect on reducing eyespot infection in the absence and presence of *Pch1* (S2 Fig). When accessing this marker’s effectiveness in lines not carrying *Pch1* it was found to cause a significant (*p* ≤ 0.005) reduction in eyespot disease, and to have even greater effect in lines with *Pch1* (S2 Fig). This region of chromosome 7B has yet to be reported in association with eyespot disease resistance. The detection in numerous environments, high phenotypic correlations, and sizeable allelic effects make this region a potential target for increasing eyespot resistance. Being that this resistance locus is on 7B, and known genes have previously been reported on 7A and 7D, this locus may be a homeoallele to *Pch1* or *Pch2*.

### Evaluating pyramiding effect of MTAs

Most MTAs discovered in this study did not appear to individually have a major effect on eyespot resistance. Therefore, we evaluated how the MTAs collectively affected disease response. By using haplotype data from the most significant tagging markers along with the *Pch1* marker data, we determined the number of eyespot resistance alleles carried by each line. A total of seven tagging markers were used for Panel A and 14 for Panel B (Tables 1 and 2). We excluded all tagging markers on chromosomes 7DL and 7AL, and markers with unknown map positions that appeared to be linked to *Pch1* or *Pch2*.

**Fig 4:**
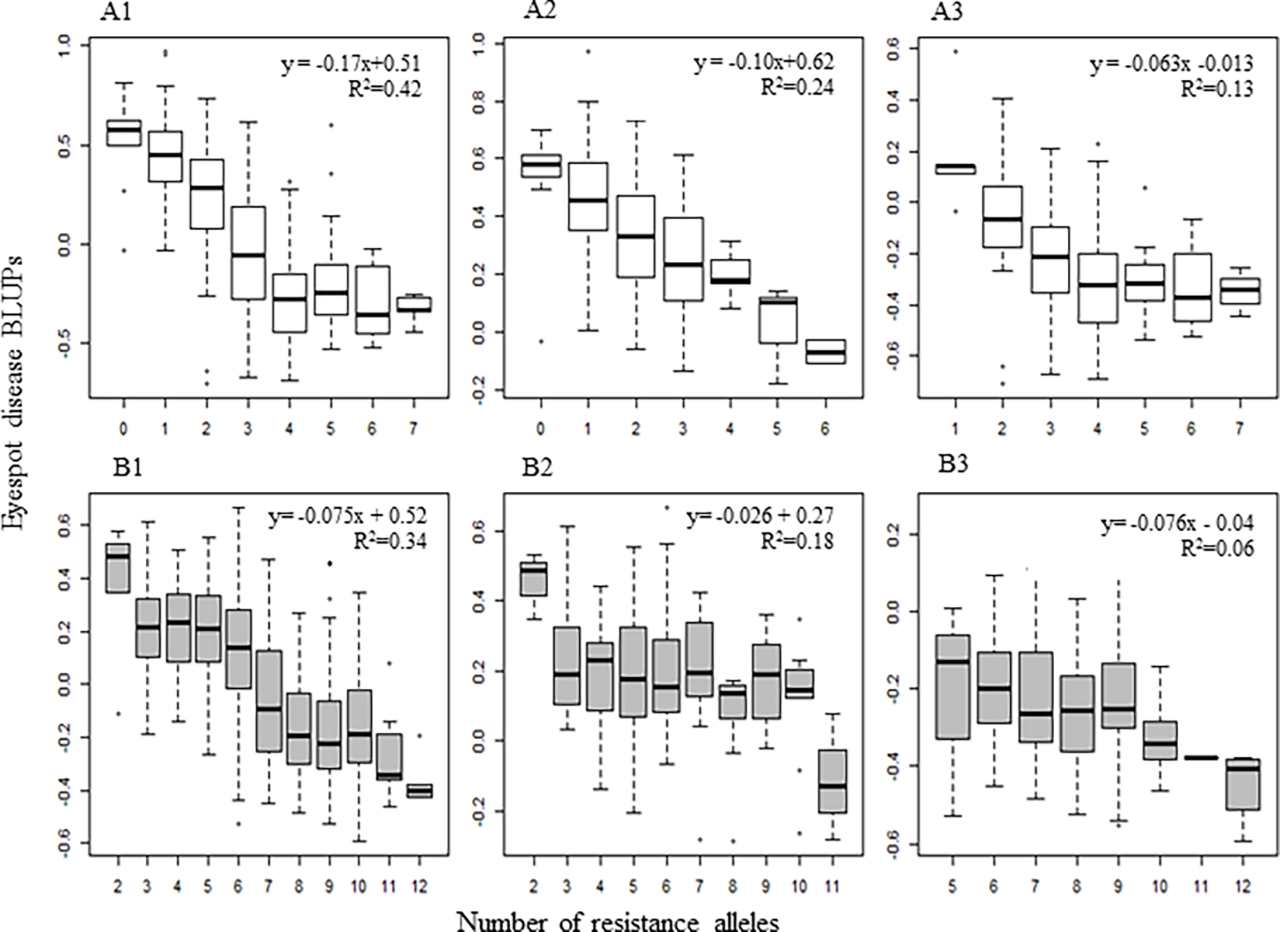
Boxplots of eyespot disease BLUPs for the number of resistance alleles for the most significant tagging markers in all winter wheat lines in Panel A (469 lines) (A1), lines in Panel A without the *Pch1* (164) (A2) and lines in Panel A with *Pch1* (253) (A3); all lines in Panel B (399) (B1), lines in Panel B without *Pch1* (143) (B2) and Panel B lines with *Pch1* (168) (B3).

The number of cumulative resistance alleles was then regressed against the combined BLUP score for all five environments. The minimum number of resistance alleles for Panels A was zero and B was two, and the maximum number was seven and twelve, respectively. The regression analysis results showed a significant effect (*p* ≤ 0.001) on eyespot disease response when the tagging markers were evaluated collectively. As the number of cumulative resistance alleles increased, in both Panels A and B, the eyespot disease scores decreased, with regression coefficients of -0.17 and -0.075 and *R*^*2*^ values of 0.42 and 0.34, respectively (Fig 4). In Panel A lines predicted to have zero resistance alleles had a mean BLUP score of 0.54, and lines with seven resistance alleles had a mean BLUP score of -0.33. The resistant controls Madsen and Cara carried four and six resistance alleles, and had very low BLUP scores of -0.48 and -0.46, respectively. On the other hand, the susceptible check Eltan carried only one resistance allele and had a high BLUP score of 0.97. In Panel B, lines predicted to have two resistance alleles had a mean BLUP score of 0.50, and lines with 12 resistance alleles had a mean score of -0.30. The resistance controls Madsen and Cara carried seven and ten resistance alleles with BLUP scores of -0.23 and -0.30. Conversely, the susceptible control Eltan had the minimum number of resistance alleles, two, and the highest BLUP score of 0.53.

To more accurately determine the effect of the minor MTAs discovered in this study two additional linear regression analyses were run, one with all lines not carrying the *Pch1* resistance allele, and one with all lines carrying the *Pch1* resistance allele. The regression analyses for lines without *Pch1* revealed a significant reduction (*p* ≤ 0.001) in the eyespot disease score as the number of cumulative resistance alleles increased, with a regression coefficient of -0.10 and an *R*^*2*^ value of 0.24 for Panel A (Fig 4). In Panel B, there was a less significant reduction (regression coefficient -0.026 and *R*^*2*^ of 0.09) in the overall disease score when resistance alleles increased (Fig 4). It appeared that lines with two resistance alleles are significantly less effective at reducing the disease score than lines with three resistance alleles. However, not until eleven cumulative resistance alleles were pyramided together did another significant (*p* ≤ 0.001) reduction in the eyespot disease response occur. The minor MTAs discovered in this study could explain most of the phenotypic variation for eyespot resistance seen in the adapted PNW winter wheat germplasm that is not explain the by the major *Pch1* resistance allele. In addition, in the analysis for all lines carrying *Pch1* there was a significant reduction (*p* ≤ 0.01) in the disease score as the number of cumulative resistant alleles increased (Fig 3). The regression coefficients for Panels A and B were -0.06 and -0.07 with *R*^*2*^ values of 0.13 and 0.06, respectively.

As advanced breeding lines and released cultivars were used in this study to identify new sources of eyespot resistance, the processes of introgressing resistance alleles into elite lines, gene pyramiding to increase resistance and improve durability, and identifying diagnostic markers can be more rapidly achieved [46]. We identified seven MTAs, including tagging markers *IWB8331*, *IWB73709*, *IWB472981*, *IWB47160*, *WB45005*, *Ex_c44379_2509*, and *IAAV4340*, which cumulatively reduced eyespot disease. We identified over thirty lines in this study that had five or more resistance allele haplotypes, and of these four were cultivars, including Cara (PI 643435), ‘Chukar’ (PI 628641), ‘Crystal’ (PI 351960), and ‘Ladd’ (OR2070870), all with six resistance alleles. Additionally, breeders may also use the significant SNP markers identified in this study to design assays for possible screening for resistant material in their own breeding programs.

## Conclusion

There are only two known sources of genetic resistance, *Pch1* and *Pch2*, currently used in US wheat breeding programs, but neither confers complete resistance. In the United State *Pch1* is the most effective and extensively used source of resistance. However, as we found and as reported by others [17, 20, 22], lines that carry *Pch1* have significantly different levels of resistance ranging from moderately susceptible to highly resistant. In addition, lines that did not carry *Pch1* had varying eyespot disease scores ranging from highly susceptible to resistant (Fig 3). Our GWAS results showed that in fact there are many minor MTAs that cumulatively contribute to eyespot resistance and explained most of the phenotypic variation not accounted for by *Pch1* or *Pch2*. The most significant MTAs identified in this study were located on chromosomes 2A, 5A, 5B, 7AS and 7B. Additionally, SNP markers associated in the same genetic region as *Pch2* were identified, although further validation is required. The significant loci discovered here will play a contributing role in the better understanding of the genetic architecture of eyespot resistance. It will also help facilitate the development of elite cultivars with stable and increased resistance when combined with *Pch1* or *Pch2*.

## Supporting information

S1 Fig Principal component analysis of Panel A using SNP genotyping data. Principal component 1 (PC1) and Principal component 2 (PC2) separated club wheat (circles) from common wheat accessions (triangles).
(DOCX)

S2 Fig Boxplots of the seven most significant tagging markers effect on eyespot BLUP scores (a) in all lines; (b) lines without *Pch1* resistance allele; and (c) all lines with the *Pch1* resistance allele. 0 = Panel A lines without resistance alleles; 1 = Panel A lines with the resistance haplotype.
(DOCX)

S1 Table Marker coverage, chromosome length, and marker density for winter wheat Panels A and B.
(DOCX)

S2 Table Pearson correlations of eyespot scores for (a) Panel A (469 lines) for five environments and BLUPs and (b) for Panel B (399 lines) for five environments and BLUPs. Panels A and B were evaluated for eyespot resistance in a total of three field environments from 2014 to 2015, and two growth chamber (GC) environments. Field locations included Washington State University (WSU) Spillman Agronomy Farm (SP) and WSU Cook Agronomy Farm (C), both located near Pullman, WA. Growth chamber environments were separated by species *O*. *acuformis* and *O*. *yullundae* (GC_OA, GC_OY).
(DOCX)

S3 Table All significant markers associated with eyespot disease resistance that were detected in two or more environments or BLUPs in winter wheat Panel A and Panel B.
(DOCX)
