# Supplementary material for: Genome-wide association mapping for eyespot disease in US Pacific Northwest winter wheat
Source: PLoS One. 2018 Apr 2;13(4):e0194698. doi: 10.1371/journal.pone.0194698 (PMC5880388; doi:10.1371/journal.pone.0194698)

**S1 Fig.** Principal component analysis of Panel A using SNP genotyping data. Principal component 1 (PC1) and Principal component 2 (PC2) separated club wheat (circles) from common wheat accessions (triangles).


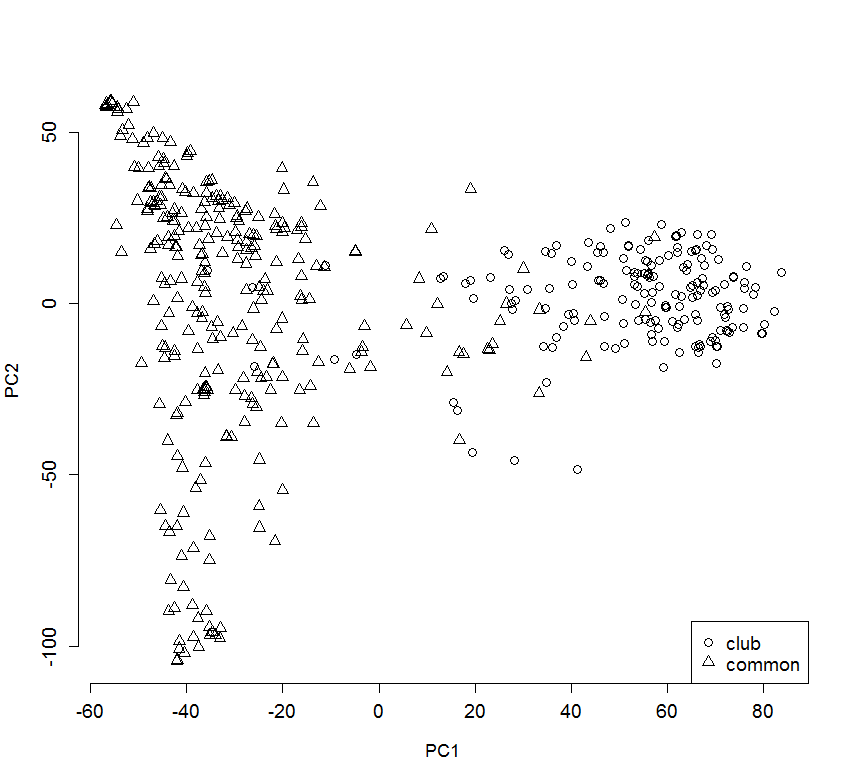

Supplement: S1 Fig — Principal component analysis of Panel A using SNP genotyping data. Principal component 1 (PC1) and Principal component 2 (PC2) separated club wheat (circles) from common wheat accessions (triangles). (DOCX) [file pone.0194698.s001.docx]
