# Supplementary material for: Genome-wide association mapping for eyespot disease in US Pacific Northwest winter wheat
Source: PLoS One. 2018 Apr 2;13(4):e0194698. doi: 10.1371/journal.pone.0194698 (PMC5880388; doi:10.1371/journal.pone.0194698)
Supplement: S2 Fig — Boxplots of the seven most significant tagging markers effect on eyespot BLUP scores (a) in all lines; (b) lines without Pch1 resistance allele; and (c) all lines with the Pch1 resistance allele. 0 = Panel A lines without resistance alleles; 1 = Panel A lines with the resistance haplotype. (DOCX) [file pone.0194698.s002.docx]

***2A_IWB8331***


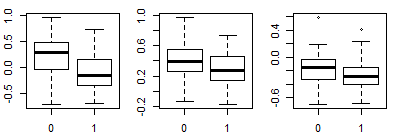


(a) (b) (c)

BLUP score

***5A_IWB73709***


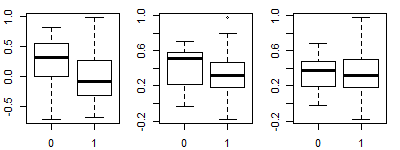


(a) (b) (c)

BLUP score

**5B_*IWB47298***


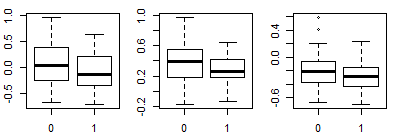


(a) (b) (c)

BLUP score


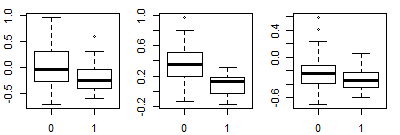
**7A_*IWB47160***

(a) (b) (c)

BLUP score

**7B_*IWB45005***


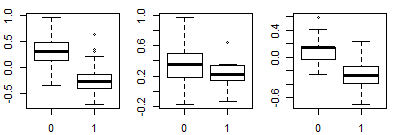


(a) (b) (c)

BLUP score

*
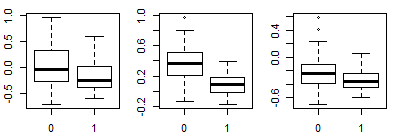
****no map position_20731***

(a) (b) (c)

BLUP score

***
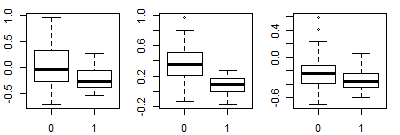
no map position_32948***

(a) (b) (c)

BLUP score

**S2 Fig.** Boxplots of the seven most significant tagging markers effect on eyespot BLUP scores (a) in all lines; (b) lines without *Pch1* resistance allele; and (c) all lines with the *Pch1* resistance allele. 0= Panel A lines without resistance alleles; 1=Panel A lines with the resistance haplotype.
