# Supplementary material for: Genome-wide association mapping for eyespot disease in US Pacific Northwest winter wheat
Source: PLoS One. 2018 Apr 2;13(4):e0194698. doi: 10.1371/journal.pone.0194698 (PMC5880388; doi:10.1371/journal.pone.0194698)
Supplement: S1 Table — Marker coverage, chromosome length, and marker density for winter wheat Panels A and B. (DOCX) [file pone.0194698.s003.docx]

| **Panel A Chromosomes** | **# of Markers** | **90K Chr. Length** | **Panel B Chromosomes** | **# of Markers** | **9K Chr. Length** |
| --- | --- | --- | --- | --- | --- |
| 1A | 1629 | 161.35 | 1A | 559 | 183.56 |
| 1B | 1949 | 174.10 | 1B | 357 | 141.59 |
| 1D | 534 | 200.37 | 1D | 101 | 145.46 |
| 2A | 1464 | 185.46 | 2A | 435 | 231.30 |
| 2B | 2130 | 188.87 | 2B | 662 | 272.78 |
| 2D | 783 | 144.50 | 2D | 107 | 191.83 |
| 3A | 1278 | 207.28 | 3A | 409 | 172.69 |
| 3B | 1681 | 153.50 | 3B | 426 | 196.43 |
| 3D | 360 | 156.06 | 3D | 34 | 84.96 |
| 4A | 1211 | 166.72 | 4A | 381 | 208.02 |
| 4B | 860 | 119.45 | 4B | 161 | 125.10 |
| 4D | 102 | 170.43 | 4D | 23 | 60.31 |
| 5A | 1324 | 148.30 | 5A | 434 | 195.33 |
| 5B | 2232 | 219.77 | 5B | 540 | 227.44 |
| 5D | 287 | 208.71 | 5D | 49 | 54.05 |
| 6A | 1468 | 180.74 | 6A | 452 | 218.15 |
| 6B | 1787 | 127.54 | 6B | 430 | 154.48 |
| 6D | 236 | 160.50 | 6D | 52 | 73.33 |
| 7A | 1553 | 244.16 | 7A | 435 | 193.61 |
| 7B | 1426 | 178.86 | 7B | 281 | 169.37 |
| 7D | 263 | 226.87 | 7D | 31 | 52.73 |
| not mapped | 4222 | - | not mapped | 424 | - |
| *Total (mapped):* | **24557** |  | *Total (mapped):* | **6359** |  |
| *Total (not mapped):* | **28779** |  | *Total (not mapped):* | **6783** |  |

**S1 Table.** Marker coverage, chromosome length, and marker density for winter wheat Panels A and B.
