# Supplementary material for: Genome-wide association mapping for eyespot disease in US Pacific Northwest winter wheat
Source: PLoS One. 2018 Apr 2;13(4):e0194698. doi: 10.1371/journal.pone.0194698 (PMC5880388; doi:10.1371/journal.pone.0194698)
Supplement: S2 Table — Pearson correlations of eyespot scores for (a) Panel A (469 lines) for five environments and BLUPs and (b) for Panel B (399 lines) for five environments and BLUPs. Panels A and B were evaluated for eyespot resistance in a total of three field environments from 2014 to 2015, and two growth chamber (GC) environments. Field locations included Washington State University (WSU) Spillman Agronomy Farm (SP) and WSU Cook Agronomy Farm (C), both located near Pullman, WA. Growth chamber environments were separated by species O. acuformis and O. yullundae (GC_OA, GC_OY). (DOCX) [file pone.0194698.s004.docx]

**S2 Table.** Pearson correlations of eyespot scores for (a) Panel A (469 lines) for five environments and BLUPs and (b) for Panel B (399 lines) for five environments and BLUPs. Panels A and B were evaluated for eyespot resistance in a total of three field environments from 2014 to 2015, and two growth chamber (GC) environments. Field locations included Washington State University (WSU) Spillman Agronomy Farm (SP) and WSU Cook Agronomy Farm (C), both located near Pullman, WA. Growth chamber environments were separated by species *O*. *acuformis* and *O. yullundae* (GC_OA, GC_OY).

| **(a)** | **SP2015** | **C2015** | **GC_OA** | **GC_OY** | **BLUPs** |
| --- | --- | --- | --- | --- | --- |
| **SP2014** | 0.559 | 0.557 | 0.535 | 0.481 | 0.762 |
| **SP2015** | - | 0.500 | 0.461 | 0.450 | 0.734 |
| **C2015** | - | - | 0.570 | 0.546 | 0.790 |
| **GC_Oa** | - | - | - | 0.741 | 0.833 |
| **GC_Ya** | - | - | - | - | 0.830 |
| **(b)** | **SP2015** | **C2015** | **GC_OA** | **GC_OY** | **BLUPs** |
| **SP2014** | 0.274 | 0.472 | 0.430 | 0.460 | 0.728 |
| **SP2015** | - | 0.360 | 0.297 | 0.169 | 0.579 |
| **C2015** | - | - | 0.470 | 0.466 | 0.749 |
| **GC_OA** | - | - | - | 0.707 | 0.805 |
| **GC_OY** | - | - | - | - | 0.830 |
