# Supplementary material for: Genome-wide association mapping for eyespot disease in US Pacific Northwest winter wheat
Source: PLoS One. 2018 Apr 2;13(4):e0194698. doi: 10.1371/journal.pone.0194698 (PMC5880388; doi:10.1371/journal.pone.0194698)
Supplement: S3 Table — All significant markers associated with eyespot disease resistance that were detected in two or more environments or BLUPs in winter wheat Panel A and Panel B. (DOCX) [file pone.0194698.s005.docx]

**S3 Table.** All significant markers associated with eyespot disease resistance that were detected in two or more environments or BLUPs in winter wheat Panel A and Panel B.

| ***SNP^a^*** | **Chr^b^** | **cM^c^** | **Minor** |  | **Environments-Panel A** | | | | | |
| --- | --- | --- | --- | --- | --- | --- | --- | --- | --- | --- |
|  |  |  | **allele^d^** |  | **BLUPs** | **SP2014** | **SP2015** | **C2015** | **GC_OA** | **GC_OY** |
| *IWA5047* | 1A | 107.66 | C | *p^e^* | 1.74E-03 | - | - | - | - | - |
|  |  |  |  | *R^2f^* | 0.01 | - | - | - | - | - |
|  |  |  |  | *AE^g^* | -0.073 | - | - | - | - | - |
| ***IWB8331*** | 2AS | 101.97 | G | *p* | 1.13E-03 | - | - | 1.72E-03 | - | - |
| *IWB43628* |  |  |  | *R^2^* | 0.01 | - | - | 0.02 | - | - |
| *IWA3569* |  |  |  | *AE* | -0.121 | - | - | -0.157 | - | - |
| *IWB2840* | 2AS | 106.30 | C | *p* | - | - | - | 1.90E-03 | - | - |
| *IWB10760* |  |  |  | *R^2^* | - | - | - | 0.02 | - | - |
|  |  |  |  | *AE* | - | - | - | -0.186 | - | - |
| *IWB71497* | 2A | 141.66 | T | *p* | - | - | - | - | 1.54E-03 | 6.37E-04 |
|  |  |  |  | *R^2^* | - | - | - | - | 0.01 | 0.02 |
|  |  |  |  | *AE* | - | - | - | - | 0.085 | 0.118 |
| *IWA5697* | 2B | 80.77 | T | *p* | 1.34E-03 | - | - | 1.47E-03 | - | 1.40E-03 |
|  |  |  |  | *R^2^* | 0.01 | - | - | 0.02 | - | 0.01 |
|  |  |  |  | *AE* | 0.055 | - | - | 0.125 | - | 0.117 |
| *IWB9450* | 2B | 81.75 | T | *p* | 1.11E-03 | - | - | 1.88E-03 | - | - |
|  |  |  |  | *R^2^* | 0.01 | - | - | 0.02 | - | - |
|  |  |  |  | *AE* | -0.081 | - | - | -0.157 | - | - |
| *IWB47345* | 4AS | 35.34 | T | *p* | - | - | - | - | - | 9.31E-04 |
|  |  |  |  | *R^2^* | - | - | - | - | - | 0.02 |
|  |  |  |  | *AE* | - | - | - | - | - | -0.268 |
| *IWA4569* | 4AS | 39.94 | G | *p* | 2.81E-03 | - | - | - | - | - |
|  |  |  |  | *R^2^* | 0.01 | - | - | - | - | - |
|  |  |  |  | *AE* | -0.103 | - | - | - | - | - |
| ***IWB73709*** | 5A | 89.02 | T | *p* | 1.94E-03 | - | - | - | - | - |
|  |  |  |  | *R^2^* | 0.01 | - | - | - | - | - |
|  |  |  |  | *AE* | 0.107 | - | - | - | - | - |
| *IWA3391* | 5A | 129.86 | T | *p* | - | 1.76E-03 | - | - | - | 1.99E-03 |
| *IWB59054* |  |  |  | *R^2^* | - | 0.02 | - | - | - | 0.01 |
|  |  |  |  | *AE* | - | -0.166 | - | - | - | -0.097 |
| *IWB34332* | 5B | 100.64 | G | *p* | 1.35E-03 | 1.32E-03 | - | - | - | - |
| *IWA6671* |  |  |  | *R^2^* | 0.02 | 0.02 | - | - | - | - |
|  |  |  |  | *AE* | -0.064 | -0.130 | - | - | - | - |
| ***IWB47298*** | 5B | 100.64 | T | *p* | 4.95E-04 | 2.04E-03 | - | - | 1.20E-03 | - |
|  |  |  |  | *R^2^* | 0.01 | 0.02 | - | - | 0.02 | - |
|  |  |  |  | *AE* | -0.080 | -0.127 | - | - | -0.112 | - |
| *IWB14635* | 5B | 104.23 | G | *p* | 1.63E-03 | 7.61E-04 | - | - | - | 1.49E-03 |
|  |  |  |  | *R^2^* | 0.01 | 0.01 | - | - | - | 0.02 |
|  |  |  |  | *AE* | -0.101 | -0.167 | - | - | - | -0.152 |
| *IWB40925* | 5B | 104.23 | T | *p* | 6.28E-04 | 4.58E-04 | - | 1.69E-03 | - | - |
| *IWB40926* |  |  |  | *R^2^* | 0.01 | 0.02 | - | 0.02 | - | - |
|  |  |  |  | *AE* | 0.070 | 0.130 | - | 0.143 | - | - |
| *IWB25936* | 5B | 104.55 | G | *p* | 3.87E-04 | 8.86E-04 | - | - | - | - |
|  |  |  |  | *R^2^* | 0.02 | 0.02 | - | - | - | - |
|  |  |  |  | *AE* | -0.088 | -0.123 | - | - | - | - |
| *IWB10471* | 7AS | 126.40 | G | *p* | 2.02E-03 | - | - | - | - | 1.04E-03 |
|  |  |  |  | *R^2^* | 0.01 | - | - | - | - | 0.01 |
|  |  |  |  | *AE* | -0.082 | - | - | - | - | -0.173 |
| ***IWB47160*** | 7AS | 126.40 | T | *p* | - | - | - | - | 5.95E-04 | 1.18E-03 |
| *IWB49474* |  |  |  | *R^2^* | - | - | - | - | 0.02 | 0.01 |
|  |  |  |  | *AE* | - | - | - | - | -0.151 | -0.186 |
| *IWB34932* | 7AS | 130.27 | T | *p* | 1.42E-03 | - | - | - | 3.92E-04 | 4.61E-04 |
|  |  |  |  | *R^2^* | 0.01 | - | - | - | 0.01 | 0.01 |
|  |  |  |  | *AE* | -0.078 | - | - | - | -0.106 | -0.134 |
| *IWB62310* | 7AL | 208.71 | G | *p* | 5.13E-06 | 7.42E-06 | - | 5.37E-05 | 7.46E-05 | - |
|  |  |  |  | *R^2^* | 0.02 | 0.04 | - | 0.03 | 0.02 | - |
|  |  |  |  | *AE* | 0.128 | 0.214 | - | 0.241 | 0.166 | - |
| *IWB25121* | 7AL | 212.66 | G | *p* | 1.38E-05 | 3.51E-05 | - | - | 3.21E-05 | 1.61E-05 |
| *IWB6268* |  |  |  | *R^2^* | 0.02 | 0.03 | - | - | 0.03 | 0.03 |
| *IWB27807* |  |  |  | *AE* | -0.157 | -0.257 | - | - | -0.224 | -0.300 |
| *IWB38552* | 7AL | 219.59 | T | *p* | 2.40E-04 | - | - | - | 3.00E-05 | 1.34E-05 |
|  |  |  |  | *R^2^* | 0.02 | - | - | - | 0.03 | 0.03 |
|  |  |  |  | *AE* | 0.103 | - | - | - | 0.177 | 0.236 |
| *IWB66765* | 7AL | 227.73 | G | *p* | 1.47E-18 | 6.24E-08 | 6.57E-05 | 9.24E-12 | 3.94E-17 | 2.15E-15 |
| *IWB23319* |  |  |  | *R^2^* | 0.10 | 0.05 | 0.06 | 0.09 | 0.11 | 0.09 |
| *IWB48849* |  |  |  | *AE* | 0.184 | 0.188 | 0.203 | 0.301 | 0.260 | 0.317 |
| *IWB23316* |  |  |  |  |  |  |  |  |  |  |
| *IWB5878* | 7AL | 232.74 | T | *p* | 2.94E-08 | 7.35E-06 | 1.77E-03 | 1.68E-04 | 1.73E-08 | 2.80E-08 |
|  |  |  |  | *R^2^* | 0.04 | 0.04 | 0.03 | 0.03 | 0.05 | 0.04 |
|  |  |  |  | *AE* | 0.118 | 0.161 | 0.164 | 0.168 | 0.180 | 0.227 |
| *IWB41099* | 7AL | 241.40 | G | *p* | 1.00E-04 |  |  |  | 1.60E-04 | 1.39E-05 |
|  |  |  |  | *R^2^* | 0.04 |  |  |  | 0.05 | 0.06 |
|  |  |  |  | *AE* | -0.130 |  |  |  | -0.260 | -0.230 |
| *IWB500* | 7B | 139.97 | T | *p* | 4.68E-04 | 2.85E-04 | - | 2.86E-04 | 8.14E-04 | 4.84E-04 |
|  |  |  |  | *R^2^* | 0.01 | 0.02 | - | 0.02 | 0.02 | 0.02 |
|  |  |  |  | *AE* | 0.109 | 0.200 | - | 0.245 | 0.155 | 0.209 |
| *IWB8911* | 7B | 145.29 | G | *p* | 7.83E-05 | 1.80E-05 | - | 4.10E-04 | 7.40E-05 | 2.81E-04 |
|  |  |  |  | *R^2^* | 0.02 | 0.03 | - | 0.02 | 0.02 | 0.02 |
|  |  |  |  | *AE* | -0.077 | -0.147 | - | -0.151 | -0.117 | -0.138 |
| *IWB11950* | 7BL | 150.60 | G | *p* | 7.47E-07 | 4.04E-05 | - | 1.44E-05 | 3.25E-07 | 2.80E-06 |
| *IWB345* |  |  |  | *R^2^* | 0.03 | 0.03 | - | 0.03 | 0.04 | 0.03 |
|  |  |  |  | *AE* | -0.101 | -0.143 | - | -0.189 | -0.157 | -0.185 |
| *IWB65383* | 7BL | 152.00 | T | *p* | 1.12E-07 | 2.50E-06 | - | 4.40E-05 | 1.08E-06 | 2.34E-05 |
|  |  |  |  | *R^2^* | 0.03 | 0.04 | - | 0.03 | 0.03 | 0.02 |
|  |  |  |  | *AE* | -0.116 | -0.171 | - | -0.185 | -0.157 | -0.176 |
| ***IWB45005*** | 7BL | 158.98 | T | *p* | 5.41E-18 | 9.18E-10 | - | 2.82E-12 | 9.40E-19 | 2.18E-12 |
| *IWB9330* |  |  |  | *R^2^* | 0.09 | 0.07 | - | 0.09 | 0.12 | 0.07 |
|  |  |  |  | *AE* | -0.187 | -0.225 | - | -0.320 | -0.285 | -0.289 |
| *IWB39063* | 7B | 162.53 | T | *p* | 1.09E-13 | 4.00E-06 | 9.61E-04 | 4.53E-11 | 1.14E-12 | 1.82E-11 |
|  |  |  |  | *R^2^* | 0.07 | 0.04 | 0.04 | 0.08 | 0.08 | 0.06 |
|  |  |  |  | *AE* | 0.152 | 0.160 | 0.173 | 0.290 | 0.218 | 0.268 |
| *IWB34231* | 7B | 163.17 | G | *p* | 1.08E-15 | 4.19E-08 | 1.20E-04 | 1.26E-10 | 3.94E-14 | 2.25E-11 |
|  |  |  |  | *R^2^* | 0.08 | 0.05 | 0.05 | 0.08 | 0.09 | 0.06 |
|  |  |  |  | *AE* | 0.175 | 0.195 | 0.203 | 0.292 | 0.242 | 0.277 |
| *IWB6281* | 7B | 164.24 | T | *p* | 8.44E-06 | 4.05E-04 | - | 7.23E-04 | 3.83E-05 | 1.56E-04 |
|  |  |  |  | *R^2^* | 0.02 | 0.02 | - | 0.02 | 0.02 | 0.02 |
|  |  |  |  | *AE* | -0.096 | -0.142 | - | -0.165 | -0.134 | -0.159 |
| *IWB12696* | 7B | 165.40 | G | *p* | 1.12E-17 | 2.19E-09 | 6.45E-05 | 4.26E-12 | 3.51E-17 | 2.97E-11 |
|  |  |  |  | *R^2^* | 0.09 | 0.06 | 0.06 | 0.09 | 0.11 | 0.06 |
|  |  |  |  | *AE* | -0.190 | -0.216 | -0.209 | -0.319 | -0.276 | -0.279 |
| *IWB58840* | 7B | 167.27 | T | *p* | 2.06E-17 | 1.35E-06 | 2.49E-04 | 9.41E-12 | 1.19E-17 | 5.01E-14 |
|  |  |  |  | *R^2^* | 0.09 | 0.04 | 0.05 | 0.09 | 0.11 | 0.08 |
|  |  |  |  | *AE* | 0.170 | 0.164 | 0.185 | 0.292 | 0.257 | 0.292 |
| *IWB16803* | 7D | 190.77 | G | *p* | 2.26E-08 | 2.21E-06 | - | 2.17E-06 | 7.88E-08 | 5.29E-07 |
|  |  |  |  | *R^2^* | 0.04 | 0.04 | - | 0.04 | 0.04 | 0.04 |
|  |  |  |  | *AE* | -0.124 | -0.173 | - | -0.217 | -0.175 | -0.213 |
| *IWB18673* | 7D | 191.07 | T | *p* | 3.85E-09 | 5.69E-06 | - | 1.67E-07 | 1.45E-08 | 9.66E-08 |
| *IWB15904* |  |  |  | *R^2^* | 0.04 | 0.04 | - | 0.05 | 0.05 | 0.04 |
|  |  |  |  | *AE* | -0.145 | -0.180 | - | -0.263 | -0.204 | -0.251 |
| *IWB34018* | 7D | 192.95 | G | *p* | 2.92E-08 | 5.80E-06 | - | 6.42E-06 | 2.53E-08 | 1.43E-06 |
|  |  |  |  | *R^2^* | 0.04 | 0.04 | - | 0.04 | 0.05 | 0.03 |
|  |  |  |  | *AE* | 0.122 | 0.165 | - | 0.205 | 0.180 | 0.202 |
| *IWB34126* | 7D | 193.20 | T | *p* | 8.75E-08 | 1.43E-06 | - | 3.88E-06 | 3.32E-07 | 2.14E-06 |
| *IWB16121* |  |  |  | *R^2^* | 0.03 | 0.04 | - | 0.04 | 0.04 | 0.03 |
| *IWB30203* |  |  |  | *AE* | -0.118 | -0.177 | - | -0.211 | -0.166 | -0.201 |
| *IWB34125* |  |  |  |  |  |  |  |  |  |  |
| *IWB34127* |  |  |  |  |  |  |  |  |  |  |
| *IWB20242* | 7DL | 194.05 | C | *p* | 3.92E-05 | 1.16E-04 | - | 8.98E-05 | 1.97E-04 | 1.50E-03 |
|  |  |  |  | *R^2^* | 0.02 | 0.03 | - | 0.03 | 0.02 | 0.01 |
|  |  |  |  | *AE* | 0.083 | 0.137 | - | 0.172 | 0.113 | 0.125 |
| *IWB34165* | 7DL | 196.96 | G | *p* | 5.17E-08 | 5.01E-07 | - | 1.68E-06 | 3.48E-06 | 1.52E-04 |
|  |  |  |  | *R^2^* | 0.04 | 0.04 | - | 0.04 | 0.03 | 0.02 |
|  |  |  |  | *AE* | 0.117 | 0.183 | - | 0.218 | 0.148 | 0.155 |
| *IWB62090* | 7DL | 197.58 | G | *p* | 7.34E-09 | 6.12E-07 | 4.77E-04 | 9.48E-06 | 8.36E-08 | 1.54E-05 |
| *IWB15958* |  |  |  | *R^2^* | 0.04 | 0.04 | 0.04 | 0.04 | 0.04 | 0.03 |
| *IWB17602* |  |  |  | *AE* | 0.126 | 0.182 | 0.186 | 0.203 | 0.175 | 0.180 |
| *IWB26781* | 7D | 202.54 | T | *p* | 1.40E-08 | 3.02E-06 | 3.87E-04 | 4.97E-05 | 8.50E-07 | 6.78E-07 |
|  |  |  |  | *R^2^* | 0.04 | 0.04 | 0.04 | 0.03 | 0.04 | 0.03 |
|  |  |  |  | *AE* | -0.124 | -0.170 | -0.191 | -0.186 | -0.161 | -0.209 |
| *IWB47130* | 7D | 203.58 | C | *p* | 6.16E-08 | 7.72E-06 | 5.32E-04 | 7.93E-05 | 1.60E-06 | 1.57E-06 |
| *IWB7301* |  |  |  | *R^2^* | 0.03 | 0.04 | 0.04 | 0.03 | 0.03 | 0.03 |
|  |  |  |  | *AE* | -0.118 | -0.163 | -0.186 | -0.181 | -0.156 | -0.201 |
| *IWB4994* | 7D | 206.75 | G | *p* | 2.69E-12 | 9.31E-07 | - | 3.53E-09 | 7.09E-11 | 1.42E-09 |
| *IWB28327* |  |  |  | *R^2^* | 0.06 | 0.04 | - | 0.07 | 0.06 | 0.05 |
|  |  |  |  | *AE* | -0.154 | -0.177 | - | -0.269 | -0.212 | -0.254 |
| *IWB6751* | 7DL | 208.10 | G | *p* | 1.15E-17 | 6.39E-09 | 1.78E-05 | 3.74E-12 | 3.86E-16 | 5.93E-11 |
| *IWB11097* |  |  |  | *R^2^* | 0.09 | 0.06 | 0.07 | 0.09 | 0.10 | 0.06 |
| *IWB11947* |  |  |  | *AE* | -0.186 | -0.206 | -0.222 | -0.315 | -0.261 | -0.269 |
| *IWB11948* |  |  |  |  |  |  |  |  |  |  |
| *IWB49080* |  |  |  |  |  |  |  |  |  |  |
| *IWB74668* | 7D | 211.90 | G | *p* | 4.29E-22 | 9.69E-10 | 1.34E-04 | 7.60E-12 | 1.80E-22 | 1.50E-17 |
|  |  |  |  | *R^2^* | 0.12 | 0.07 | 0.05 | 0.09 | 0.15 | 0.11 |
|  |  |  |  | *AE* | -0.214 | -0.225 | -0.206 | -0.314 | -0.322 | -0.360 |
| *IWB18349* | 7D | 226.87 | G | *p* | 2.41E-21 | 9.20E-09 | 1.11E-04 | 3.46E-13 | 3.30E-20 | 1.89E-15 |
|  |  |  |  | *R^2^* | 0.11 | 0.06 | 0.05 | 0.10 | 0.13 | 0.09 |
|  |  |  |  | *AE* | 0.220 | 0.221 | 0.225 | 0.354 | 0.316 | 0.349 |
| *IWB5931* | - | - | G | *p* | 2.39E-19 | 2.97E-09 | 1.32E-04 | 1.26E-13 | 1.38E-17 | 3.76E-12 |
|  |  |  |  | *R^2^* | 0.10 | 0.06 | 0.05 | 0.11 | 0.11 | 0.07 |
|  |  |  |  | *AE* | -0.226 | -0.236 | -0.225 | -0.378 | -0.314 | -0.329 |
| *IWB6836* | - | - | T | *p* | 1.99E-11 | 3.14E-06 | - | 2.72E-09 | 2.09E-10 | 5.23E-08 |
|  |  |  |  | *R^2^* | 0.05 | 0.04 | - | 0.07 | 0.06 | 0.04 |
|  |  |  |  | *AE* | 0.157 | 0.176 | - | 0.285 | 0.219 | 0.242 |
| *IWB12202* | - | - | G | *p* | 3.47E-19 | 1.96E-09 | 1.75E-04 | 2.34E-13 | 6.41E-18 | 1.08E-11 |
|  |  |  |  | *R^2^* | 0.10 | 0.06 | 0.05 | 0.10 | 0.11 | 0.07 |
|  |  |  |  | *AE* | -0.224 | -0.240 | -0.223 | -0.375 | -0.317 | -0.322 |
| *IWB14903* | - | - | G | *p* | 2.29E-10 | 1.82E-06 | - | 7.44E-08 | 4.31E-09 | 3.29E-08 |
|  |  |  |  | *R^2^* | 0.05 | 0.04 | - | 0.05 | 0.05 | 0.04 |
|  |  |  |  | *AE* | -0.149 | -0.181 | - | -0.260 | -0.203 | -0.248 |
| ***IWB20731*** | - | - | G | *p* | 8.53E-04 | - | - | - | - | 7.59E-04 |
|  |  |  |  | *R^2^* | 0.01 | - | - | - | - | 0.02 |
|  |  |  |  | *AE* | -0.084 | - | - | - | - | -0.164 |
| *IWB26205* | - | - | G | *p* | 1.11E-08 | 3.36E-06 | - | 6.07E-07 | 4.06E-08 | 6.99E-07 |
|  |  |  |  | *R^2^* | 0.04 | 0.04 | - | 0.05 | 0.04 | 0.03 |
|  |  |  |  | *AE* | -0.135 | -0.179 | - | -0.243 | -0.190 | -0.224 |
| ***IWB32948*** | - | - | G | *p* | 3.70E-04 | - | - | - | 3.23E-04 | 4.68E-04 |
|  |  |  |  | *R^2^* | 0.01 | - | - | - | 0.02 | 0.02 |
|  |  |  |  | *AE* | 0.103 | - | - | - | 0.152 | 0.193 |
| *IWB34988* | - | - | T | *p* | 1.45E-09 | 7.73E-07 | 1.84E-03 | 5.01E-06 | 1.07E-07 | 6.30E-08 |
|  |  |  |  | *R^2^* | 0.04 | 0.04 | 0.03 | 0.04 | 0.04 | 0.04 |
|  |  |  |  | *AE* | 0.141 | 0.187 | 0.174 | 0.218 | 0.183 | 0.241 |
| *IWB34862* | - | - | G | *p* | 1.26E-03 | - | - | - | - | - |
|  |  |  |  | *R^2^* | 0.01 | - | - | - | - | - |
|  |  |  |  | *AE* | -0.099 | - | - | - | - | - |
| *IWB42932* | - | - | G | *p* | 5.18E-08 | 1.46E-06 | - | 7.01E-07 | 2.40E-06 | 7.12E-05 |
|  |  |  |  | *R^2^* | 0.04 | 0.04 | - | 0.05 | 0.03 | 0.02 |
|  |  |  |  | *AE* | 0.122 | 0.181 | - | 0.234 | 0.156 | 0.170 |
| *IWB48221* | - | - | T | *p* | 8.52E-12 | 2.91E-08 | - | 1.61E-06 | 1.02E-08 | 9.37E-09 |
|  |  |  |  | *R^2^* | 0.06 | 0.06 | - | 0.04 | 0.05 | 0.05 |
|  |  |  |  | *AE* | -0.149 | -0.203 | - | -0.220 | -0.186 | -0.240 |
| *IWB49786* | - | - | T | *p* | 5.15E-10 | 3.09E-08 | - | 6.99E-07 | 2.11E-06 | 2.41E-07 |
|  |  |  |  | *R^2^* | 0.05 | 0.05 | - | 0.05 | 0.03 | 0.04 |
|  |  |  |  | *AE* | 0.137 | 0.212 | - | 0.234 | 0.156 | 0.218 |
| ***SNP^a^*** | **Chr^b^** | **cM^c^** | **Minor** |  | **Environments-Panel B** | | | | | |
|  |  |  | **allele^d^** |  | **BLUPs** | **SP2014** | **SP2015** | **C2015** | **GC_OA** | **GC_OY** |
| ***IWA5505*** | 1AL | 118.27 | G | *p^e^* | 3.96E-03 | 6.63E-04 | - | - | - | - |
| *IWA4934* |  |  |  | *R^2f^* | 0.02 | 0.03 | - | - | - | - |
|  |  |  |  | *AE* | -0.063 | -0.261 | - | - | - | - |
| ***IWA4897*** | 1AL | 137.20 | T | *p* | 5.41E-04 | 1.38E-04 | - | - | - | - |
|  |  |  |  | *R^2^* | 0.03 | 0.03 | - | - | - | - |
|  |  |  |  | *AE* | -0.137 | -0.149 | - | - | - | - |
| ***IWA4089*** | 1BL | 78.45 | G | *p* | 9.92E-04 | - | 8.93E-06 | - | 2.16E-04 | 1.37E-04 |
|  |  |  |  | *R^2^* | 0.02 | - | 0.04 | - | 0.03 | 0.03 |
|  |  |  |  | *AE* | -0.111 | - | -0.212 | - | -0.212 | -0.286 |
| ***IWA5161*** | 2A | 167.87 | G | *p* | 8.97E-06 | 8.61E-05 | - | 1.87E-03 | 5.10E-04 | 9.75E-04 |
|  |  |  |  | *R^2^* | 0.04 | 0.03 | - | 0.02 | 0.02 | 0.02 |
|  |  |  |  | *AE* | -0.068 | -0.042 | - | -0.108 | -0.089 | -0.070 |
| ***IWA551*** | 2DL | 98.59 | T | *p* | 3.13E-04 | - | - | - | 1.34E-03 | 1.42E-03 |
| *IWA5894* |  |  |  | *R^2^* | 0.03 | - | - | - | 0.02 | 0.02 |
|  |  |  |  | *AE* | 0.096 | - | - | - | 0.119 | 0.186 |
| ***IWA4054*** | 3B | 62.57 | C | *p* | - | - | - | - | 1.67E-03 | 1.40E-03 |
|  |  |  |  | *R2* | - | - | - | - | 0.02 | 0.02 |
|  |  |  |  | *AE* | - | - | - | - | -0.136 | -0.114 |
| ***IWA8203*** | 3B | 144.74 | T | p | - | - | - | - | 5.12E-06 | 7.01E-04 |
| *IWA2147* |  |  |  | *R^2^* | - | - | - | - | 0.04 | 0.03 |
|  |  |  |  | AE | - | - | - | - | -0.126 | -0.121 |
| ***IWA5923*** | 5A | 15.86 | G | *p* | 1.46E-03 | 1.83E-03 | - | - | - | - |
| *IWA3567* |  |  |  | *R^2^* | 0.02 | 0.02 | - | - | - | - |
|  |  |  |  | *AE* | 0.068 | 0.046 | - | - | - | - |
| ***IWA1*** | 5AL | 90.54 | G | p | - | - | - | 1.39E-03 | - | 1.26E-03 |
| *IWA4719* |  |  |  | *R^2^* | - | - | - | 0.02 | - | 0.02 |
|  |  |  |  | AE | - | - | - | -0.276 | - | -0.288 |
| ***IWA7708*** | 5B | 150.93 | G | *p* | 1.07E-03 | - | - | - | - | - |
|  |  |  |  | *R^2^* | 0.02 | - | - | - | - | - |
|  |  |  |  | *AE* | -0.052 | - | - | - | - | - |
| ***IWA8312*** | 7AL | 171.11 | T | *p* | 2.33E-04 | - | - | - | 5.21E-04 | 1.98E-04 |
|  |  |  |  | *R^2^* | 0.03 | - | - | - | 0.02 | 0.03 |
|  |  |  |  | *AE* | 0.109 | - | - | - | 0.174 | 0.246 |
| ***IWA598*** | 7B | 142.24 | G | *p* | 1.26E-06 | 5.24E-04 | - | 5.04E-04 | 2.36E-05 | 1.65E-05 |
|  |  |  |  | *R^2^* | 0.05 | 0.03 | - | 0.03 | 0.04 | 0.04 |
|  |  |  |  | *AE* | -0.087 | -0.148 | - | -0.043 | -0.106 | -0.170 |
| ***IWA505*** | - | - | G | *p* | - | - | - | - | 3.01E-04 | 4.84E-04 |
|  |  |  |  | *R^2^* | - | - | - | - | 0.03 | 0.02 |
|  |  |  |  | *AE* | - | - | - | - | 0.113 | 0.089 |
| ***IWA4046*** | - | - | C | *p* |  | - | - | - | 4.78E-04 | 1.59E-03 |
|  |  |  |  | *R^2^* | - | - | - | - | 0.03 | 0.02 |
|  |  |  |  | *AE* | - | - | - | - | -0.292 | -0.172 |
| ***IWA2226*** | - | - | G | *p* | 1.65E-03 | - | - | - | 1.48E-03 | 4.91E-04 |
|  |  |  |  | *R^2^* | 0.02 | - | - | - | 0.02 | 0.03 |
|  |  |  |  | *AE* | -0.067 | - | - | - | -0.094 | -0.162 |

| ^a^ Underlined SNP markers indicates ‘tagging marker’, bold indicates ‘most significant tagging marker’ |
| --- |
| ^b^ ‘-’ indicates unmapped SNPs that were significant in this analysis |
| ^c^ Chromosome positon according to Wang et al. (2014) |
| ^d^ Allele that the allelic effect estimate (AE) is in respect to |
| ^e^ Indicates the significance of SNP marker |
| ^f^ R^2^indicates phenotypic variation explained by significant SNP |
| ^g^ AE is the allelic effect estimate in respect to the minor allele |
